# Proteostasis unbalance in prion diseases: Mechanisms of neurodegeneration and therapeutic targets

**DOI:** 10.3389/fnins.2022.966019

**Published:** 2022-09-06

**Authors:** Stefano Thellung, Alessandro Corsaro, Irene Dellacasagrande, Mario Nizzari, Martina Zambito, Tullio Florio

**Affiliations:** ^1^Section of Pharmacology, Department of Internal Medicine (DiMI), University of Genova, Genova, Italy; ^2^IRCCS Ospedale Policlinico San Martino, Genova, Italy

**Keywords:** prion protein, protein misfolding, autophagy, proteasome, neurodegeneration

## Abstract

Transmissible spongiform encephalopathies (TSEs), or prion diseases, are progressive neurodegenerative disorders of the central nervous system that affect humans and animals as sporadic, inherited, and infectious forms. Similarly to Alzheimer's disease and other neurodegenerative disorders, any attempt to reduce TSEs' lethality or increase the life expectancy of affected individuals has been unsuccessful. Typically, the onset of symptoms anticipates the fatal outcome of less than 1 year, although it is believed to be the consequence of a decades-long process of neuronal death. The duration of the symptoms-free period represents by itself a major obstacle to carry out effective neuroprotective therapies. Prions, the infectious entities of TSEs, are composed of a protease-resistant protein named prion protein scrapie (PrP^Sc^) from the prototypical TSE form that afflicts ovines. PrP^Sc^ misfolding from its physiological counterpart, cellular prion protein (PrP^C^), is the unifying pathogenic trait of all TSEs. PrP^Sc^ is resistant to intracellular turnover and undergoes amyloid-like fibrillation passing through the formation of soluble dimers and oligomers, which are likely the effective neurotoxic entities. The failure of PrP^Sc^ removal is a key pathogenic event that defines TSEs as proteopathies, likewise other neurodegenerative disorders, including Alzheimer's, Parkinson's, and Huntington's disease, characterized by alteration of proteostasis. Under physiological conditions, protein quality control, led by the ubiquitin-proteasome system, and macroautophagy clears cytoplasm from improperly folded, redundant, or aggregation-prone proteins. There is evidence that both of these crucial homeostatic pathways are impaired during the development of TSEs, although it is still unclear whether proteostasis alteration facilitates prion protein misfolding or, rather, PrP^Sc^ protease resistance hampers cytoplasmic protein quality control. This review is aimed to critically analyze the most recent advancements in the cause-effect correlation between PrP^C^ misfolding and proteostasis alterations and to discuss the possibility that pharmacological restoring of ubiquitin-proteasomal competence and stimulation of autophagy could reduce the intracellular burden of PrP^Sc^ and ameliorate the severity of prion-associated neurodegeneration.

## Introduction

The capacity to withstand environmental variations, keeping a continuous control of structure and function, represents a primary need for all living beings and is often referred to as homeostasis. In both uni- and multicellular organisms, an extraordinarily wide range of mechanisms cooperate to keep homeostasis, whose functioning is a mandatory requirement to grant long-term survival (Magalhaes et al., 2018). Protein homeostasis, or proteostasis, balances protein synthesis and degradation not only to adapt the number of intracellular proteins to the cell needs but also to control their proper folding, trafficking, and recycling; this is particularly relevant within the central nervous system (CNS) to prolong neuronal viability, synaptic functions, and ultimately cognitive performances (Hetz, 2021). Defective proteostasis is the basis of CNS alterations in several pathologic conditions. Protein misfolding, oligomer/fibryl accumulation, and cellular inability to eliminate them are the common histopathological traits of Alzheimer's disease (AD), Parkinson's disease (PD), fronto-temporal dementia (FTD), Huntington's disease (HD), amyotrophic lateral sclerosis (ALS), and transmissible spongiform encephalopathies (TSEs), also known as prion diseases (Thellung et al., 2019; Le Guerroue and Youle, 2021). Walker and Levine III (2000) proposed the term *cerebral proteopathy* to semantically unify all the disorders that are associated with the alteration of protein conformation in the CNS. Such semantic unification is particularly useful because comprehends, under a common basic alteration, neurodegenerative diseases otherwise heterogeneous for etiology, histopathology, and clinical presentation. Although sporadic onset represents the most prevalent occurrence of all neurodegenerative disorders, significant signs of progress in the characterization of the mechanisms of pathogenesis have been obtained by studying inherited forms of proteopathies. Noteworthy, pathogenic mutations have been described not only in genes encoding for disease-specific proteins but also in genes involved in the mechanisms of protein quality control and turnover (Lehman, 2009). TSEs, rare and fatal neurodegenerative conditions universally known as prion diseases, affect humans and domestic and feral animals, inducing a rapid neurological decline. Neurohistology lesions, although differences among the different forms are present, comprise spongiform degeneration of brain parenchyma, neuronal loss, astrogliosis, and deposition of the amyloidogenic protein, prion protein scrapie (PrP^Sc^). PrP^Sc^ is derived from the post-translational rearrangement of the glycoprotein cellular prion protein (PrP^C^) (Prusiner and Dearmond, 1991). Human TSEs, including Creutzfeldt–Jakob disease (CJD), Gerstmann–Sträussler–Scheinker disease (GSS), fatal familial insomnia (FFI), and Kuru, are different in etiology being sporadic, familial, iatrogenic, and infectious. A peculiar distinctive feature of TSEs is transmissibility through inter-human passage of PrP^Sc^ (Prusiner, 1998; Will and Ironside, 2017). PrP^C^ to PrP^Sc^ transition may be either spontaneous or favored by mutations within *PRNP*, the PrP^C^ encoding gene, or forced by the interaction with exogenous PrP^Sc^ that acts as a template (Prusiner, 1982). PrP^Sc^ infection may follow parenteral or enteral routes (for example, via ingestion of contaminated meat during the “mad cow” disease epidemic in the 1990s; Diack et al., 2014). PrP^C^ misfolding leading to the formation of PrP^Sc^ concerns the secondary structure of the protein, increasing β-sheet structure content, hydrophobicity, and resistance to proteolysis (Pan et al., 1993). PrP^Sc^ accumulation leads to the activation of amyloidogenic pathways, leading to the formation of oligomers and fibrils, whose accumulation is generally associated with histological lesions in all prion diseases (Corsaro et al., 2012).

Although a direct cause-effect relationship between PrP^Sc^ accumulation and pathology is still a debated issue (Parchi et al., 1996; Hill and Collinge, 2003), there is growing evidence that PrP^C^ misfolding is normally prevented by a protein quality control system whose failure plays a pathogenic role in all TSEs. Moreover, the impairment of the two major cellular mechanisms that control proteostasis, the ubiquitin–proteasome quality control (UPS), and autophagy (ALP) has been also described in association with other neurodegenerative disorders characterized by protein misfolding (Nixon, 2013; Ciechanover, 2015; Zheng et al., 2016; Colacurcio et al., 2018). These similar pathogenic mechanisms, identified in all proteopathies, prompted the search for common targets to Exeter effective neuroprotective intervention in all these conditions (Kumar et al., 2020). This review is focused on the most recent insights into the pathogenic role of proteostasis failure in prion diseases and the possibility to counteract neuronal death through the pharmacological modulation of UPS and autophagy.

## Protein quality control

In eukaryotic cells, the control of proper folding of newly synthesized proteins and the prevention of their aberrant accumulation are granted by a series of interconnected mechanisms, whose intervention ranges from assisted refolding in the endoplasmic reticulum (ER), to degradation or disaggregation in the cytosol. All these pathways provide vital contributions to proteostasis and are also joined in a complex network of mutually compensatory activities (Ciechanover and Kwon, 2017). Improper protein folding often produces the exposure of hydrophobic amino acid sequences that are recognized and bound by chaperones resident in ER, nucleus, and cytoplasm. Binding to chaperones favors the reestablishment of the correct structure of client proteins or drives them to proteolysis *via* the ubiquitin-proteasomal system (UPS) or the autophagic pathway. There is compelling evidence that cellular and pathological prion proteins are crucial clients of the mechanisms of protein quality control undergoing chaperone-assisted refolding (Tittelmeier et al., 2020), ubiquitination, proteasomal, and lysosomal degradation (Ciechanover, 2015). There is also hope that a better comprehension of these pathways may lead to novel effective neuroprotective strategies against prion diseases.

### Chaperone-assisted refolding and proteolysis

Molecular chaperones is a large group of evolutionarily conserved proteins that play a vital role in protein quality control and allow adaptations of the cellular proteome to environmental changes; their loss of efficacy in CNS has often been associated with brain proteopathies (Ciechanover and Kwon, 2017).

Chaperones assist proper protein folding by recognizing exposed hydrophobic sequences in nascent polypeptides as a hallmark of protein misfolding and forming complexes that temporarily block proteins in an unfolded state. This complexation may either determine the correct refolding of client proteins or activate their degradation by the ubiquitin-proteasome system (UPS) and autophagy. Heath shock proteins (HSPs) are chaperones activated by a variety of cell stresses, including heat, oxidative, and inflammatory insults; they comprehend different subgroups (named based on respective molecular weight: Hsp90, HsP70, Hsp40, and others). Accessory proteins, named co-chaperones, are also often recruited to the complexes with substrate proteins and act as adaptors to guide ubiquitination and interaction with proteasomes (Abildgaard et al., 2020). Not surprisingly, the control of prion protein folding is an important task of molecular chaperones and is currently under investigation to characterize its involvement in prion disease pathogenesis, as well as the therapeutic potential of its pharmacological modulation (Tittelmeier et al., 2020; Thackray et al., 2022).

### Ubiquitin proteasomal system

Eukaryotic cells rely on UPS for survival because this proteolytic system exerts a continuous control of the quality and the amount of newly formed, short-living proteins. Excessive and/or wrongly folded proteins, instead of being transported from rough ER to Golgi, for further processing and insertion in the plasma membrane or secretion, are retrotranslocated in the cytosol, conjugated with multiple chains of ubiquitin, and degraded by the 26S proteasome (Pickart, 2001; Sloper-Mould et al., 2001). The addition of poly-ubiquitin chains, essential for further proteolytic processing of the substrate, is carried out by a sequence of three ATP-consuming steps, catalyzed by the ubiquitin-associated enzymes E1, E2, and E3. Ubiquitin is transiently bound to and activated by E1 (ubiquitin-activating enzyme), transferred to E2 (ubiquitin-conjugating enzyme), and then to E3 (ubiquitin ligase) that covalently binds ubiquitin to substrates. This process is repeated several times by adding further units to the previously attached ubiquitin, thus tagging the substrates by a chain of poly-ubiquitin that is recognized by 26S proteasome (Glickman and Ciechanover, 2002). Proteasome is a large cytosolic and nuclear 26S, ATP-dependent enzymatic complex, composed of a catalytic (proteolytic) 20S unit and a regulatory 19S unit, both composed of multiple subunits. The regulatory 19S unit that forms a cap at one or both sides of the 20S unit, comprehends a base and a lid and works as a gatekeeper that selects the substrate to degrade and prepare it for digestion. Broadly, the 19S unit recognizes ubiquitinylated proteins, removes the poly-ubiquitin chains, and unfolds the proteins, thus allowing the recycling of ubiquitin moieties and the entry of the substrates into the 20S proteolytic chamber (Sahu and Glickman, 2021). The catalytic 20S subunit has the shape of a cylinder, composed of four stacked rings, each made of seven subunits; the two external rings are called α rings and delimitate the entrance and exit of the barrel, whereas the two β rings, endowed with caspase-like, trypsin-like, and chymotrypsin-like proteolytic activity, digest the proteins (Glickman and Ciechanover, 2002; Sakata et al., 2021). While digestion is carried on by β subunits, the α-structures control the opening of the gate to let the substrate in and expel digested peptides.

The extremely complex regulation of ubiquitin ligation and proteasome functioning is beyond the aim of this review, and thus, we will focus on the most recent insight into the association between UPS failure and the development of neurodegeneration in prion diseases.

### Autophagy

Autophagy is a lysosomal-dependent, pro-survival proteolysis of portions of cytoplasm, damaged organelles, and potentially harmful protein, which allows cells to cope with several stress conditions. Through autophagy, cells may survive starvation *via* the recycling of cytoplasmic nutrients, avoid apoptosis by removing damaged mitochondria, and control proteostasis by digesting aberrant and aggregation-prone proteins (Klionsky et al., 2021).

Different types of autophagy can be identified based on the nature of substrates and the mechanisms by which substrates are delivered to lysosomes. While macroautophagy, classically referred to as autophagy, traps large portions of cytoplasm in double-membrane autophagosomes that fuse with lysosomes to digest and recycle nutrients, other protein-targeted forms called aggrephagy and chaperone-mediated autophagy (CMA) play a crucial role in the control of proteostasis.

Aggrephagy, cooperates with UPS, being mainly involved in the removal of larger or aggregation-prone proteins that cannot enter the narrow pore of the proteasome (Lamb et al., 2013; Kumar et al., 2022). Broadly, misfolded proteins that escape the degradation by proteasome because of abundancy, aggregated structure, or as a consequence of defects of specific UPS steps, cluster in the cytoplasm to form inclusion bodies (Kopito, 2000), which may be engulfed in autophagosomes (Fortun et al., 2003). Aggrephagy is a remarkably selective process, allowed by adaptor proteins in which clusters of ubiquitinylated substrates committed to degradation are driven into growing phagophores and digested as autophagosomes fused with lysosomes (Bjorkoy et al., 2005; Pankiv et al., 2007). Perhaps the best characterized of these adaptors is a multidomain protein of 440 amino acids, named sequestosome 1 (SQSTM1) or p62, whose activity is not exclusively involved in the proteostasis, but lies at the crossroads of multiple signaling (Katsuragi et al., 2015). P62 role has been extensively studied in many neurodegenerative diseases, and its accumulation on the cytoplasm is nowadays regarded as a reliable hallmark of either activation of autophagy or reduced efficiency of a protein quality control system (Pankiv et al., 2007; Klionsky et al., 2021). The sequence of p62 has been defined in detail, leading to the identification of multiple domains responsible for protein-to-protein interactions (Berkamp et al., 2021). Three of these domains are considered critical to govern the fate of protein degradation between UPS and aggrephagy: the PB1 domain that allows p62 oligomerization, the LIR domain that interacts with LC3BII located in the membranes of phagophores, and a ubiquitin-binding domain (UBA) that binds ubiquitinylated substrates (Pankiv et al., 2007; Katsuragi et al., 2015). There is recent evidence that condensation of cytoplasmic proteins in solid aggregates, as occurs in typical aggrephagy, is not the only mechanism by which autophagosomes eliminate clustered misfolded proteins. Through its UBA domain, p62 sequesters ubiquitinylated proteins into membrane-less droplets, called p62 bodies that increase in size by progressive coalescence (Berkamp et al., 2021). Although composed of aggregated proteins, p62 bodies have a spherical shape and a viscosity that separates them from cytosol with a liquid-liquid phase separation (Brangwynne et al., 2009; Sun et al., 2018). Through LIR sequence, p62 interacts with LC3-II proteins that marks the growing phagophore, thus promoting the engulfment of the bodies in the autophagosome (Zaffagnini et al., 2018; Simonsen and Wollert, 2022). The process continues with the fusion of autophagosomes with lysosomes, allowing the digestion and the recycling of ubiquitinylated substrates present in the autophagosomes (Klionsky et al., 2021).

Selective removal of proteins from cytosol is made possible by an alternative form of autophagy called chaperone-mediated autophagy (CMA), in which client substrates bind with chaperone and co-chaperone complexes and are translocated within lysosomes by lysosome-associated protein 2A (LAMP2A) (Bourdenx et al., 2021). Typical targets of CMA contain the amino acid sequence KFERQ and need the binding of chaperones belonging to the family of Hsp70 and the heat shock protein 70 cognate (Hsc70) that are overexpressed under stressful conditions. Nevertheless, other chaperones, linked to the control of the cell cycle, have been described to operate *via* CMA recognizing different pentapeptide sequences, for example, in prion proteins (Wang et al., 2015, 2017).

## Protein quality control of prion protein

PrP^C^ is a glycoprotein anchored to the outer leaflet of the plasma membrane *via* a glycosylphosphatydylinositol moiety (Stahl et al., 1987). Along the pathway of PrP^C^ maturation, the removal of the N-terminal signal peptide is followed by the formation of a disulfide bridge and the addition of one or two oligosaccharide chains (Haraguchi et al., 1989). GPI anchor is hence attached to the N-terminus of the protein following the removal of a 22 AA peptide (Stahl et al., 1992; Hegde and Rane, 2003). Mature PrP^C^ is exposed on the outside of the plasma membrane surface and, being characterized by a high turnover and rapidly recycled through the endocytic system (Shyng et al., 1993), it is a likely candidate to undergo UPS control. PrP^C^ maturation follows the typical route for transmembrane proteins as the removal of signal peptide and glycosylation in the ER, translocation to Golgi, and the insertion into the outer leaflet of the plasma membrane.

Crucial scientific questions about the biology of PrP^C^ have pertained to the molecular partners that not only assist its proper folding but also affect PrP^C^-PrP^Sc^ conversion or bring misfolded/aggregated prions to degradation. Among the several chaperones reported to interact with prion proteins under physiological and pathological circumstances, those belonging to heat shock proteins and in particular the Hsp70 subgroup (Hsp70s) showed a consistent association with prion diseases in humans, animals, and in experimentally infected rodents and cells. Indeed, the analysis of brains from sporadic and familial CJD patients showed that Hsp73 expression is inversely proportional to neuronal loss indicating that differences of vulnerability to PrP^Sc^ in different brain areas may depend on the neuroprotective activity of chaperones (Kovacs et al., 2001). Similar reports have been obtained in scrapie-infected sheep where the pattern of expression of heat shock proteins was associated with prion protein deposition, gliosis, and spongiosis (Serrano et al., 2011). In mice infected by intracerebral injection of scrapie strains, Hsp70 immunoreactivity was detected in close vicinity of lysosome-related structures (Laszlo et al., 1992) and a net increase of Hsp70 and ubiquitin gene expression was observed (Kenward et al., 1994). Pharmacological induction of Hsp70 in chronically prion-infected cell lines counteracted intracellular accumulation of PrP^Sc^, and, on the other hand, the abrogation of Hsp70 in mice accelerates the progression of neurodegeneration after the intracerebral injection of RML prion (Mays et al., 2019). Another molecular chaperone, whose activity plays a role in the posttranslational fate of prion protein, is the 78 KDa glucose-regulated protein (GPR78), also known as binding immunoglobulin protein (Bip), a member of HsP70s, mainly resident in the ER. Independent researchers have demonstrated that Bip can bind physiological and disease-associated forms of PrP, inducing proteasomal degradation of mutant PrP (Jin et al., 2000; Park et al., 2017). Importantly, Bip can also revert PrP^Sc^ protease resistance and can inhibit PrP^Sc^ replication in persistently infected cells, and its expression is necessary to prolong mouse survival after intracerebral infection with scrapie strains (Park et al., 2017). Moreover, although devoid of consequence in normal conditions, knocking out stress-sensing proteins, such as heath shock factor 1, reduces survival in mice infected with scrapie prions (Steele et al., 2008). Altogether these reports suggest that activation of chaperones belonging to the group of Hsps represents an attempt of neuroprotection that may explain the late onset of most familial TSEs.

PrP^C^ is a typically short-lived protein with a half-life of about 6 h (Taraboulos et al., 1992), and it is estimated that about 10% of newly formed protein does not pass the quality control and is degraded by the proteasome. In fact, pharmacological inhibition of proteasome activity produces an accumulation of cytosolic aggregates of protease-resistant PrP, rich in ubiquitin moieties (Ma and Lindquist, 2001; Yedidia et al., 2001), whereas the inhibition of proteasome negative regulators causes a reduction of the PrP^Sc^ intracellular content (Homma et al., 2015). Prion protein mutations are associated with familial forms of CJD, GSS, and FFI, producing PrP variants characterized by incomplete processing and altered folding and topology. The degradation of these mutant prion proteins is partially granted by chaperones that temporarily bind the protein favoring its processing by UPS, CMA, or disaggregation (Jin et al., 2000; Wang et al., 2017; Thackray et al., 2022). *In vitro* studies, performed in cell lines expressing PrP mutant forms, have demonstrated that the pharmacological inhibition of UPS induces intracellular accumulation of detergent-insoluble and partially protease-resistant PrP that accumulates in aggregation bodies (Zanusso et al., 1999; Jin et al., 2000; Ma and Lindquist, 2001; Mishra et al., 2003). Moreover, the ubiquitinylation of PrP^C^ by the ubiquitin ligase TRAF6 favors its interaction with p62 and the formation of aggresomes (Masperone et al., 2022).

Similarly, to PrP^C^ and its variants harboring disease-associated mutations, the presence of PrP^Sc^, the pathogenic form of PrP, induces the cells to activate the UPS and favors the formation of aggregates, as a defense strategy. Using intracerebral and intraperitoneal prion injection in experimental animals as infection models, Homma observed an increased expression of p62 and autophagosome-associated LC3-II in the brain of terminally ill animals (Homma et al., 2014). Moreover, the pharmacological inhibition of proteasome, using the inhibitor MG-132, stimulated the formation of perinuclear aggregates containing both PrP^Sc^ and p62 in PrP^Sc^ persistently infected neuroblastoma cells. Importantly, p62 colocalization with PrP^Sc^ is dependent on p62 ability to bind ubiquitin. High levels of p62 also abrogate cell commitment to apoptosis when mutant forms of PrP^C^ escape UPS degradation and form PrP- and p62-containing cytoplasmic inclusion bodies (Xu et al., 2014). Although intracellular accumulation of PrP^Sc^ is believed to be counteracted by the autophagosome-autolysosome pathway, there is evidence that PrP^Sc^ can be also recognized by chaperones and digested in lysosomes through the activation of CMA. One of these chaperones is the polo-like kinase 3 (PLK3), whose overexpression upregulates Hsc70 and LAMP2A, which, in turn, translocate PrP^Sc^ in the lysosomal lumen (Wang et al., 2015, 2017). Altogether, this evidence suggests that the UPS system could, at least temporarily and partially, counteract the accumulation of pathological prion proteins in the brain, during the development of both inherited and acquired forms of TSEs and that selective aggrephagy and CMA can act as complementary clearing systems when aggregated PrP^Sc^ escape UPS or because of its overwhelming.

The evolving concept of transmissible proteopathies faced the crucial issue to define the mechanisms that may favor the diffusion of aberrant proteins, in particular prion protein, to distant body districts or within the brain. Several mechanisms have been hypothesized including axonal- or cell-mediated transport and diffusion through macrovesicles released from infected neurons (Glatzel and Aguzzi, 2000; Bellingham et al., 2012). In particular, microvesicle-mediated transport is emerging as a pivotal mechanism for short- and long-range intercellular communication. It was reported that exosomes, microvesicles contained in multivesicular bodies (MVBs), are released into the extracellular space and uptaken by neighboring cells through receptor-mediated endocytosis or phagocytosis. Importantly, both exosome release and autophagy activation are used by cells not only for cell-to-cell communication but also for proteostasis maintenance (Xu et al., 2018).

**Figure 1:**
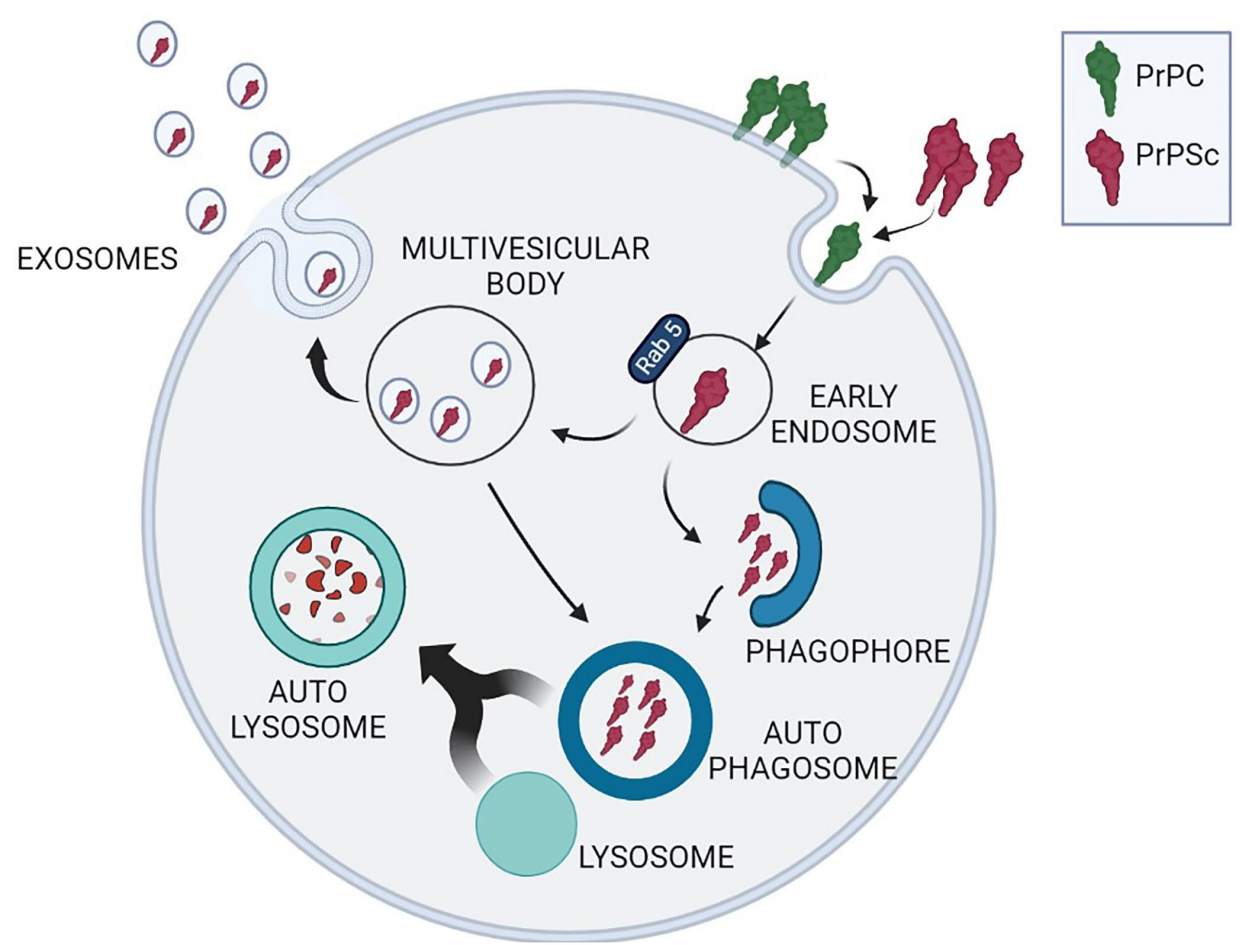
Schematic representation of the interplay between exosome release and autophagy to reduce the intracellular burden of PrP^Sc^. PrP^C^ (green) conversion into neurotoxic PrP^Sc^ isoform (red) occurs during PrP^C^ recycling through a stochastic event or after interaction with exogenous PrP^Sc^. Neuronal strategies to remove cytoplasmic PrP^Sc^ comprehend protein degradation through macroautophagy or PrP^Sc^ insertion into exosomes contained in multivesicular bodies (MVBs). MVBs can either release exosomes containing PrP^Sc^, or reenter the autophagy cycle through the formation of autophagosomes, amphysomes (not depicted in the figure) and autolysosomes.

The cooperation between autophagy and exosome release is particularly relevant for neurons that alternatively use these systems to reduce the burden of unwanted proteins. Cell commitment to degrade or release cargo proteins contained in exosomes is evidenced by the fate of MVBs: that may fuse with autophagosomes and follow the autophagy flux producing the digestion of exosomes and their cargo or they may fuse with the plasma membrane and release exosomes in the extracellular space (Fader et al., 2008) (Figure 1). Prion-infected cells release exosomes containing PrP^Sc^ which can transmit disease to recipient mice (Fevrier et al., 2004; Vella et al., 2007). These *in vitro* results are in line with the histochemical analysis of autophagy-related structures in TSE-affected brains, revealing the presence of MVBs immunoreactive for PrP^Sc^, suggesting exosome-mediated diffusion of PrP^Sc^ within the brain (Sikorska et al., 2004; Liberski et al., 2011; Guo et al., 2016).

Neuronal capacity to engulf disease-associated proteins through exosomes is currently under extensive investigation and has been described as a “double-edged sword” in many protein conformational diseases including tauopathies and α-synucleinopathies, where neurons can adopt the same strategy to avoid apoptosis while favoring the dissemination of pathology (Perez-Gonzalez et al., 2020; Meldolesi, 2021). Importantly, pharmacological modulation of autophagy is easily achievable thus producing an indirect modulation of exosome release (Abdulrahman et al., 2018; Thellung et al., 2019).

Recently, it was discovered that also other neurotoxic protein aggregates from different neurodegenerative diseases (AD and PD in particular) can be transported from affected areas to distant neurons. In particular, misfolded forms of b-amyloid or α-synuclein can be secreted into the extracellular milieu, possibly via exosomes, thereby affecting in a prion-like manner the normal physiology of the neighboring cells. This hypothesis was proposed as a plausible reason for the time course of idiopathic AD and PD, which only become symptomatic in middle to late life. For a review, see Yoshida and Hasegawa (2022).

## Proteostasis failure is a pathogenic trait shared by prion diseases and other neurodegenerative conditions

Neuronal plasticity and long-term survival rely on a complex network of sensors that detect perturbation of proteostasis and effectors that put in action a series of countersteps, including chaperone-assisted protein refolding and disaggregation, UPS, and autophagy (Weibezahn et al., 2005; True, 2006; Overhoff et al., 2021; Giandomenico et al., 2022). The loss of competence of these control systems, although occurring at a low level also during physiological aging, is enhanced in many neurodegenerative disorders and favors the accumulation of misfolded proteins, the production of neurotoxic oligomers, and their diffusion within CNS (Braak et al., 2004; Nixon, 2013; Tsakiri and Trougakos, 2015). The discovery of pathogenic prions has attracted an unprecedented interest in protein folding as a transmissible disease-related trait. This observation prompted neuroscientists to consider protein aggregation as a dynamic phenomenon in which protein tendency to adopt aberrant conformations may take over the proteolytic capacity of host cells and, under certain circumstances, favor the diffusion within the brain or be transmissible in other organisms (Harrison et al., 2001). Indeed, the Braak hypothesis, originally formulated to explain the progressive infiltration of adjacent brain areas with Levy bodies in Parkinson's disease, has been recently applied to explain also the progression of hyperphosphorylated tau within dystrophic neurons in Alzheimer's disease, and the centripetal progression of PrP^Sc^ from the periphery to the brain in infectious TSEs (Braak and Braak, 1995; Braak et al., 2004; Iwasaki, 2020). Moreover, the capacity of a pathological protein to recruit its physiological counterpart driving its spatial refolding, which originally was described as a unique property of PrP^Sc^, has been recently proposed to play a role also in the pathogenesis of Alzheimer's and Parkinson's diseases, as a mechanism favoring intracellular aggregation of hyperphosphorylated tau and α-synuclein (Caughey and Kraus, 2019; Duyckaerts et al., 2019). According to the “gain of toxicity” hypothesis, to explain the neuroanatomical alterations associated with the accumulation of PrP^Sc^ in the brain, it was proposed that, regardless of the etiology of the disease (transmission from infected material, mutation in the PrP-encoding gene, or stochastic events), neuronal death results from the toxic activity of PrP soluble oligomers generated during the amyloidogenic process occurring after its misfolding (Bucciantini et al., 2002; Chiovitti et al., 2007; Simoneau et al., 2007). The different human prion diseases are characterized by remarkable differences in clinical presentation and neuropathology, in particular as PrP^Sc^ deposits in brain parenchyma. Amyloid plaques containing PrP^Sc^ are not invariantly present, being detected in a minor part of variant and sporadic CJD cases, and their ultrastructural aspect ranges from multicentric, typical of familiar GSS, florid in most cases of variant CJD or unicentric, typical of kuru (Ghetti et al., 1995; Ironside and Bell, 1997; Sikorska et al., 2009; Liberski et al., 2010). Regardless of this heterogeneity, the presence of ubiquitin surrounding amyloid plaques and its association with dystrophic neurons in spongiotic areas has been consistently reported by histochemical and ultrastructural analyses of brains in vCJD, GSS, and kuru patients, and it is an important common trait that prion diseases share with Alzheimer's and other neurodegenerative conditions (Suenaga et al., 1990; Ironside et al., 1993; Lowe et al., 1993; Migheli et al., 1994; Sikorska et al., 2009; Zhou et al., 2015).

The increase in vacuolar structures with the features of lysosomes, containing ubiquitinylated proteins, and of large autophagosomes, containing ribosomes and other cytoplasmic constituents, has been often described by post-mortem analysis of brains from animals and humans affected by prion diseases and in experimentally prion-infected rodent brains (Boellaard et al., 1989, 1991; Jeffrey et al., 1992; Liberski et al., 1992; Alves-Rodrigues et al., 1998). Moreover, by electron microscopy observation of brain tissue from scrapie-infected brains, the presence of electron-dense lysosomes containing undigested prion protein, ubiquitin, and chaperons was observed, suggesting that the impairment of the proteolytic cycle leads to lysosomal disruption and neuronal death (Laszlo et al., 1992).

Relevant insight regarding such issue also comes from studies about the pathogenic consequence of autophagy impairment in Alzheimer-related neuronal death. Immunohistochemical analysis of cortical dystrophic neurites of patients affected by Alzheimer's disease shows a significant increase in electron-dense autophagosomes compared with age-matched healthy subjects (Boland et al., 2008). In this regard, it has been recently demonstrated that the extracellular deposition of β-amyloid-containing plaques is the consequence of impaired autophagic digestion of Aβ peptides, due to the lack of acidification of autolysosomes and incomplete autophagy cycle (Lee et al., 2022). Importantly, this alteration largely precedes extracellular deposition of β-amyloid, and it was proposed to represent a causal event in amyloid plaque formation.

Given the involvement of UPS and autophagy impairment in a broad spectrum of human proteopathies (Bence et al., 2001; Paul, 2008), it will be crucial to understand whether the intracellular aggregation of prion protein is the consequence or the cause of proteostasis disruption. Such an important issue is presently unresolved, although evidence of a causal role of autophagy impairment in protein aggregation can be derived from *in vitro* and preclinical studies, which demonstrated that the stimulation of autophagy prevents or reverts the aggregation, and the subsequent toxicity of several misfolded proteins including PrP^Sc^ or derived peptides (Cortes et al., 2012; Nakagaki et al., 2013; Thellung et al., 2019). The incubation of neuroblastoma cells with a misfolded (Corsaro et al., 2006; Chiovitti et al., 2007), neurotoxic (Thellung et al., 2013, 2017) recombinant PrP peptide was shown to impair the proteostatic machinery leading to cathepsin D leakage from lysosomes and inhibition of the normal autophagic flux that lead to cell death (Thellung et al., 2011, 2018). Moreover, It was reported that intracerebral infection with PrP^Sc^ in mice led to the deposition of ubiquitin-tagged proteins in brain areas interested by vacuolation and the deposition of amyloid plaques containing PrP^Sc^ (Lowe et al., 1990, 1992; Lopez-Perez et al., 2020). These studies also described a correlation between the severity of symptoms and the presence of ubiquitinylated proteins in the mouse brain that, already detectable within a few months post-infection, increased in quantity along with the deposition of protease-resistant PrP^Sc^ and tissue degeneration (Lowe et al., 1992; Kenward et al., 1994; Kang et al., 2004). The distribution of ubiquitin-tagged proteins also significantly colocalizes with the lysosomal enzyme cathepsin D and, as revealed by electron microscopy analysis, it is concentrated in vesicular structures, possibly lysosomes or autophagosomes (Lowe et al., 1990, 1992; Ironside et al., 1993). Moreover, in scrapie infected mice, in the late stages of the disease, an increase of p62 was observed, indicating the impairment in autophagic flux progression and a possible deficiency of neuronal lysosomal-mediated control of proteostasis (Homma et al., 2014; Lopez-Perez et al., 2020).

**Figure 2:**
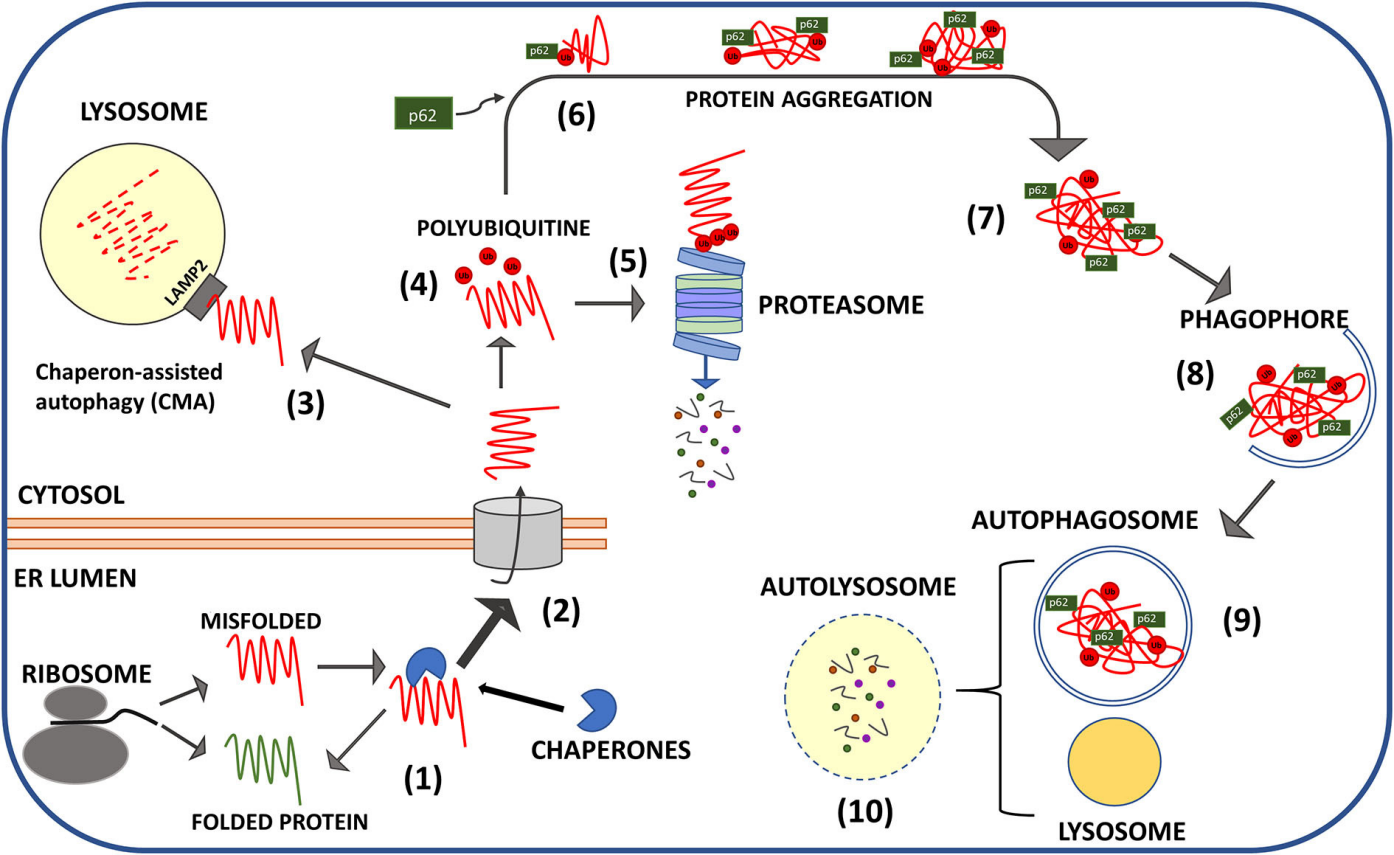
Schematic representation of protein quality control mechanisms of misfolded PrP. Structural aberrancy of nascent PrP^C^ (spontaneously occurring or favored by *PRNP* mutations) is sensed in the ER leading to the recruitment of chaperones that block PrP^C^ in unfolded state until proper folding (1) is restored. Terminally misfolded PrP is translocated in the cytosol (2) for lysosomal chaperone associate autophagy (CMA) (3) or ubiquitination (red dots: ubiquitin moieties) (4) and proteasomal digestion (5). Cytosolic aggregates of ubiquitinylated PrP escape proteasome (6) and are clustered in larger inclusion bodies by the intervention of adaptor proteins mainly p62 (7). P62 drives the aggregates toward the nascent phagophores that engulf PrP clusters in autophagosomes (8). Autophagosomes fusion with lysosomes produces digestion of aggregates (9) and recycling of nutrients (10).

Direct links between pathological prion proteins and the impairment of specific proteostatic effectors are reported by relevant papers and comprehend mainly the downregulation of chaperones in prion-infected cell lines and animals, and the impairment of the capacity of the proteasome to accept the aggregated form of PrP^Sc^ for degradation (Figure 2).

Studies performed in transgenic mice and transfected cell lines harboring mutant forms of PrP have demonstrated that *PRNP* mutations produce modifications in structure, topology, and metabolism of mature PrP and favor spontaneous conversion in protease-resistant PrP^Sc^ (Petersen et al., 1996; Capellari et al., 2000a,b; Chiesa et al., 2000; Stewart et al., 2001; Zaidi et al., 2005; Corsaro et al., 2011; Quaglio et al., 2011). Impaired proteostasis may also favor PrP^Sc^ accumulation in some late-onset inherited forms of prion diseases since it has been demonstrated that disease-associated mutations of prion protein downregulates chaperones and hamper UPS-mediated proteolysis (Zanusso et al., 1999; Peters et al., 2016). Although the capacity to avoid retrotranslocation in the cytosol and ubiquitination is not invariantly associated with PrP mutations, there is evidence that the expression of mutant prion proteins associated with familiar CJD (e.g., V210I and M323R) induces downregulation of the ER chaperone Bip and the activity of Hrd1 ubiquitin ligase (Peters et al., 2016). A heritable form of GSS, linked with an amber mutation (Y145stop) in *PRNP* gene, produces a truncated form of PrP lacking the C-terminal GPI, which retains its N-terminal signal peptide and is not properly exposed in the plasma membrane. Such form of PrP is rapidly degraded by intact UPS, but accumulates in the cytoplasm as PK-resistant protein if UPS is pharmacologically blocked (Zanusso et al., 1999; Jin et al., 2000). Of particular relevance, it has been demonstrated that both PrP^Sc^ extracted from scrapie-infected mouse brain and recombinant β-refolded prion protein inhibit the proteolytic activity of 26S proteasome due to the stabilization of the 20S core in a closed conformation and the prevention of its opening by ubiquitinylated substrates (Kristiansen et al., 2007; Deriziotis et al., 2011). Conversely, proteasome activity was not inhibited by fibrillar PrP^Sc^, and it was restored if PrP^Sc^ or β-refolded recombinant PrP are incubated with antibodies directed against its oligomeric aggregation state (Kristiansen et al., 2007).

UPS blockade, which mainly affects chymotrypsin and caspase-like proteolytic activity of the proteasome, leads to the intracellular accumulation of otherwise short-living proteins and has been proposed as a possible mechanism by which PrP^Sc^ oligomers exert neurotoxicity (Amici et al., 2010; Deriziotis et al., 2011). Robust scientific evidence links ubiquitin accumulation and proteasomal dysfunction with neuronal rarefaction in different conditions beyond TSEs. This decline occurs during aging (Carrard et al., 2002), AD (Gregori et al., 1997; Tseng et al., 2008), PD (Lindersson et al., 2004), and poly(Q) repeat-related diseases (Verhoef et al., 2002). Extensive studies have proposed that proteasomal activity may prevent intracerebral accumulation of β-amyloid peptides and hyperphosphorylated tau. Aβ binding to the 20s proteolytic unit of proteasome causes a reduction of its enzymatic activities (Gregori et al., 1995, 1997; Cecarini et al., 2008). In line with this evidence, mainly derived from cell lines or transgenic mouse studies, a reduction of proteasomal activity was also identified in post-mortem brain samples from AD patients. Proteosome activity was particularly reduced in the hippocampus and in cortical areas in which neurodegeneration is prominent, while it was not affected in the cerebellum and in the occipital cortex where tissue degeneration is usually not detectable (Keller et al., 2000). Similarly, autoptical analysis of substantia nigra of idiopathic PD patients demonstrated the presence of partially inactive proteasome indicating that, even in the absence of α-synuclein or parkin mutations, proteasome impairment contributes to the formation of Lewy bodies and nigral neurodegeneration (Mcnaught et al., 2001). More recently, soluble oligomers of Aβ1-42, α-synuclein, and poly-Q huntingtin were demonstrated to be able to block proteasome activity stabilizing the gate in the closed conformation, through a specific binding with its α subunit (Thibaudeau et al., 2018). These reports indicate that the accumulation of ubiquitinylated proteins observed in prion diseases may be caused by PrP^Sc^ itself through the impairment of proteasome-mediated protein degradation and that this mechanism represent could represent a major cause of neurotoxicity induced by misfolded oligomers in a wide range of neurodegenerative diseases.

## Pharmacological enhancement of autophagy is a therapeutic perspective for prion diseases

Except for the vCJD outbreak in the mid of 1990s that attracted a significant interest in the scientific community on prion biology, the very low incidence of human TSEs, with the most frequent sCJD affecting 1–2 individuals *per* 1,000,000 persons *per* year, poses objective obstacles toward the pursuit of a cure. The scarcity of recruitable patients and the rapid progression of neurological and cognitive decline after diagnosis are the reason of very few clinical trials performed, which were also characterized by the frustrating lack of efficacy of all the therapeutic approaches that have been so far tested. Noteworthy, most of these trials have been carried out using structurally and clinically unrelated compounds with the main goal to prevent PrP^C^-PrP^Sc^ conversion and PrP^Sc^ aggregation as observed in preclinical studies (Forloni et al., 2019).

However, the growing evidence that the impairment of proteostasis is a shared pathogenic trait between prion diseases and other protein misfolding diseases suggests the utility of changing therapeutic strategy to adopt a common approach for all neurodegenerative disorders of CNS, both the more prevalent and rarest ones (Butler et al., 2006; Nixon, 2013; Engelender et al., 2022). Significant preclinical evidence supports the idea that pharmacological stimulation of autophagy through mTOR-dependent and independent mechanisms is beneficial to reduce neuronal death and prolong survival associated with amyloidogenic proteins such as α-synuclein, Aβ, and poly(Q)-huntingtin. The molecular pathways involved are heterogeneous and comprehend the reduction of hyperactivated mTOR, the increased digestion of misfolded oligomers, and the removal of damaged mitochondria preventing Cytochrome C diffusion and activation of apoptosis (Bove et al., 2011; Bordi et al., 2019).

Newer approaches able to boost the self-defensive proteostatic competence of neurons are presently investigated (Thellung et al., 2019). Many of these compounds display a still non-characterized anti-prion activity, evidenced the ability to activate autophagy flux and have been proposed as proof of principle of common therapeutic strategy against different proteopathies of CNS with the aim of restoring proteostasis (Table 1). The first evidence in such direction has been provided by the use of macrolide derivatives, originally used as immunosuppressant drugs by the virtue of their capacity to inhibit cell cycle progression and protein synthesis, but that are also able to inhibit mTORC phosphorylation to induce autophagy (Rubinsztein et al., 2007). Among these, sirolimus (rapamycin) and tacrolimus, along with their derivatives (rapalogues) everolimus and temsirolimus, are still the most effective and powerful autophagy activators. Although no anti-prion, compassionate or off-label, use of rapamycin or rapalogues on humans has yet been reported, there is preclinical evidence that these drugs could play a role in the treatment of familial forms of prion diseases. In particular, it was demonstrated that rapamycin reduces the deposition of amyloid plaques and delays symptoms' onset in transgenic mice that express a mutant PrP (A116V), associated with an inheritable form of GSS (Cortes et al., 2012), and restored autophagy flux in neuroblastoma cells incubated with a cytotoxic recombinant PrP-derived peptide (Thellung et al., 2018). In accordance with this evidence, tacrolimus induces a sustained autophagic activity leading to the degradation of newly misfolded PrP^Sc^ molecules in persistently infected cells and counteracts PrP^Sc^ accumulation in the brain of mice intracerebrally inoculated with two prion strains. Thus, the pharmacological inhibition of mTOR could find rational use also in the treatment of non-inherited forms of prion disease (Nakagaki et al., 2013). It must be acknowledged, however, that the side effects of all rapalogues when used in a chronic regimen pose significant uncertainty about the feasibility of such therapy for neurological diseases (Mandrioli et al., 2018).

**Table 1:** Preclinical anti-prion activity of autophagy enhancers.

**Drug**	**Class**	**Mechanism of action**	**References**
Rapalogues	Immunosuppressant	mTOR inhibition	Cortes et al., 2012
Astemizole	H1 histamine receptor antagonist	mTOR inhibition	Karapetyan et al., 2013
Lithium, valproic acid	Mood stabilizers	Phosphoinositide turnover inhibition	Heiseke et al., 2009
Trehalose	Disaccharide	Autophagy activation and chaperone-like inhibition of PrP^Sc^ aggregation	Beranger et al., 2008; Aguib et al., 2009
Metformin	Hypoglycemizing	AMPK-dependent activation of autophagy	Abdelaziz et al., 2020

The proof-of-principle of a possible anti-prion activity of the autophagy-stimulating molecules, acting through the inhibition of mTOR, prompted the screening of many other compounds, heterogeneous for structure, origin, mechanism of action, and possible original therapeutic indications, for the capacity to stimulate autophagy and inhibit intracellular aggregation of PrP^Sc^ and other aggregation-prone polypeptides linked to brain proteopathies. Moreover, the possibility to stimulate autophagy without interfering with the function of immune systems, as is the case of rapamycin analogs, would be highly desirable in virtue of a potentially milder toxicity in chronic treatment. Indeed, besides the direct mTOR inhibition, other mechanisms that can stimulate autophagy and produce inhibition of PrP^Sc^ intracellular accumulation have been described.

Astemizole, an H1 histamine receptor antagonist, has the capacity to inhibit mTOR signaling and is able to prevent PrP^Sc^ replication in a persistently infected neuroblastoma cell line, prolonging the survival of mice infected with intracerebral inoculation of RML scrapie prion (Karapetyan et al., 2013; Lyu et al., 2018).

Metformin is a first-choice drug for type II diabetes that is currently repurposed for independent clinical indications (Wurth et al., 2014). Metformin exerts hypoglycemic activity through the activation of the AMP-activated protein kinase (AMPK) (Zhou et al., 2001) and displays a pro-autophagic activity through the same mechanism (Lu et al., 2016). AMPK stimulates the formation of autophagosomes by inhibiting mTOR and has been described to reduce the amount of PrP^Sc^ in a neuroblastoma cell line persistently with permanent infection (Heiseke et al., 2009; Howell et al., 2017; Abdelaziz et al., 2020).

Mood stabilizers, as lithium and valproic acid, have been recently demonstrated to possess the ability to stimulate neuronal autophagy and increase neuronal resistance to neurotoxic proteins, inhibiting Intracellular accumulation and toxicity of Aβ peptides, mutant huntingtin, and α-synuclein. The most exhaustive characterization of the pharmacodynamical basis of their neuroprotective activity is obtained with lithium that stimulates the formation of autophagosomes through the blockade of phosphoinositol turnover and IP_3_ production (Sarkar and Rubinsztein, 2006). Another promising compound is the natural disaccharide trehalose produced by bacteria, yeast, and small invertebrate, due to its capacity to stabilize protein folding (Wolkers et al., 2001). Trehalose displays neuroprotective activity against neuronal death induced by different types of aggregation-prone proteins, including Aβ, tau, huntingtin, α-synuclein, and SOD1 (Tanaka et al., 2004; Liu et al., 2005; Rodriguez-Navarro et al., 2010). Trehalose stimulates the clearance of amyloid-related oligomers, through mTOR-independent activation of autophagy flux (Tanaka et al., 2004; Sarkar et al., 2007; Mardones et al., 2016). Using persistently infected neuroblastoma cells as a model of PrP^Sc^, trehalose reduced the amount of protease-resistant PrP^Sc^ accumulated within the cells, protecting them from oxidative damage (Beranger et al., 2008; Aguib et al., 2009). Recently, it has been also proposed the efficacy of trehalose in reducing the formation of amyloid plaques since it may interfere with the formation of Aβ peptides altering the endosomal-mediated cleavage of the amyloid precursor protein (Tien et al., 2016; Liu et al., 2020).

## The present and the future of neuroprotective therapies targeting protein clearance

Four decades have passed since the first formulation of the prion hypothesis, and an apparent scientific heresy has become a major milestone for the biology of living beings and the pathology of some sporadic, familial, and infectious neurodegenerative disorders of humans and animals (Prusiner, 1982, 1998). The discovery of a physiological counterpart of the pathological PrP in the brain that under certain circumstances undergoes an aberrant refolding, had a special aftermath among neuroscientists. In particular, a great interest was posed on the role of protein misfolding in the pathogenesis of many neurodegenerative disorders characterized for the deposition of aggregated proteins in inclusion bodies, neurofibrillary tangles, and extracellular amyloid plaques (Basler et al., 1986; Scheckel and Aguzzi, 2018). Besides the limited efficacy of the non-opioid drug flupirtine in contrasting cognitive decline in CJD patients, by virtue of antiapoptotic activity (Otto et al., 2004), most of the therapeutic approaches so far evaluated for human TSEs were directed to contrast PrP^C^ misfolding by preventing its interaction with PrP^Sc^ templates. On the basis of encouraging results, obtained *in silico* or in experimentally infected rodents, a number of drugs, whose chronic administration was considered ethical, were tested as inhibitors of the PrP^C^-PrP^Sc^ transition. One of the first strategies, which has been pursued to prevent PrP^C^-PrP^Sc^ conversion or stimulate PrP^Sc^ removal, employed natural and synthetic polymers endowed with PrP^Sc^ and misfolded PrP^C^ binding ability, acting as chaperone-like agents (Teruya and Doh-Ura, 2022). Prompted by the emergency caused by the vCJD outbreak among young people in the UK in the 1990s, the encouraging results obtained by treating scrapie-infected mice with the anionic polymer pentosane polysulfate in terms of prolonging post-infection survival (Doh-Ura et al., 2004) were readily translated in human treatment programs. Few vCJD patients have been treated, by intraventricular route, with pentosane polysulfate and evidenced a significant increased survival (Newman et al., 2014). The capacity of the antimalarial drug quinacrine to interfere with the PrP^C^-PrP^Sc^ interface, by virtue of its planar three-dimensional, resulted in neuroprotection in cellular and animal models of TSEs (Forloni et al., 2002; Villa et al., 2011), and inspired different clinical trials in humans. Two double-blind, randomized trials were conducted on more than 150 patients affected by sporadic, familial, iatrogenic, and variant CJD; patients were orally treated with quinacrine and evaluated for survival, and neurological and cognitive decline (Collinge et al., 2009; Geschwind et al., 2013). Pursuing a similar strategy, other clinical trials, using tetracycline antibiotics doxycycline and minocycline, were shown to be efficacious *in vitro* (Forloni et al., 2002; Gu and Singh, 2004; De Luigi et al., 2008; Corsaro et al., 2009), and doxycycline was tested in patients affected by CJD and FFI. However, despite the encouraging preclinical effects the administration of either quinacrine or doxycycline did not produce significant effects on survival in CJD patients (Collinge et al., 2009; Haik et al., 2014; Varges et al., 2017; Forloni et al., 2019). Noteworthy, results from a clinical trial performed using doxycycline as preventive approach in FFI in still asymptomatic patients are expected in the near future (Forloni et al., 2015). In 2018, another experimental treatment program was initiated at University College London Hospital on six patients diagnosed with CJD (five sporadic and one iatrogenic) to evaluate the tolerability and efficacy of a humanized monoclonal antibody, named PRN100 directed against PrP^C^, administered by intravenous administration (Mead et al., 2022). None of the aforementioned programs have shown stable cognitive improvement or prolonged survival, although the treatment with PRN100 was very well tolerated (Table 2). A possible factor that explains this lack of efficacy is that, differently from animal models, human prion diseases are characterized by a very long asymptomatic phase in which progressive accumulation of PrP^Sc^ occurs. Thus, diagnosis and treatments start when the prion burden in the brain and neuronal death are already beyond sensitivity to any therapy (Forloni et al., 2019). Conceptually similar, disease modifying therapeutic approach aimed to delay neuronal death in Alzheimer's disease and Parkinson's disease, the most diffuse neurodegenerative conditions among elderly, are directed to the removal of Aβ peptides, hyperphosphorylated tau protein, and α-synuclein (Engelender et al., 2022). These strategies, pursued with the use of antibodies specifically directed toward Aβ, tau, and α-synuclein showed good efficacy in animal models of these diseases and led to the approval by US Food and Drug Administration of a human monoclonal antibody (aducanumab) directed against Aβ (Cummings et al., 2021). Regretfully, most efforts in developing effective immunotherapy against AD did not produce effective results and also the use of aducanumab is still debated and its actual efficacy has not been completely proved (Whitehouse et al., 2022). The increasing characterization of the mechanisms of proteostasis and the evidence of a relationship between UPS and ALP malfunctioning and accumulation of misfolded proteins is opening a new therapeutic strategy focused on restoring protein metabolism control in neurons, rather than acting on the disease-specific proteins, might pave the way for future therapeutic approaches possibly acting on all proteopathies (Gregori et al., 1995; Ciechanover and Brundin, 2003; Ciechanover and Kwon, 2017; Thellung et al., 2019; Chen et al., 2021; Leri et al., 2021).

**Table 2:** Human clinical trials.

**Compound** **(class)**	**Mechanism of action** **(hypothetical)**	**Trial/(status)**	**Disease (patients'** **number)**	**Outcome**	**References**
Flupirtine (non-opioid analgesic)	Antiapoptotic	Double-blind, randomized (closed)	sCJD (28)	Delayed cognitive decline; no extended survival	Otto et al., 2004
Quinacrine (antimalarial)	Inhibition of PrP^C^-PrP^Sc^ interaction	Patient-preference (closed)	sCJD (45) fCJD (42) vCJD (18) iCJD (2)	No effects on clinical course	Collinge et al., 2009
Quinacrine (antimalarial)	Inhibition of PrP^C^-PrP^Sc^ interaction	Double-blind placebo-controlled (Closed)	sCJD (54)	No effects on clinical course	Geschwind et al., 2013
Pentosan polysulfate (chaperone)	PrP^C^ stabilization	Observational study (Closed)	vCJD (5)	Extended survival (unclear reason)	Newman et al., 2014
Doxycycline (antibiotic)	Inhibition of PrP^C^/PrP^Sc^ interaction	Phase 2, randomized, double-blind, placebo-controlled (Closed)	CJD (121)	No effects on clinical course	Haik et al., 2014
Doxycycline (antibiotic)	Inhibition of PrP^C^/PrP^Sc^ interaction	Phase 2, randomized, double-blind, placebo-controlled (Closed)	sCJD (62)	Slight extension of survival	Varges et al., 2017
Doxycycline (antibiotic)	Inhibition of PrP^C^/PrP^Sc^ interaction	Preventive trial Patient-preference (ongoing)	FFI (25)	Ongoing	Forloni et al., 2015
PRN100 (anti-PrP^C^ monoclonal Ab)	Removal of PrP^C^	Systematic observation	sCJD (5) iCJD (1)	Ongoing	Mead et al., 2022

## Author contributions

All authors contributed to the design, draft preparation, reviewing, and editing of the manuscript. All authors contributed to the article and approved the submitted version.

## Funding

This work was supported by IRCCS Ospedale Policlinico San Martino Ricerca Corrente 2022 grant to TF.

## Conflict of interest

The authors declare that the research was conducted in the absence of any commercial or financial relationships that could be construed as a potential conflict of interest.

## Publisher's note

All claims expressed in this article are solely those of the authors and do not necessarily represent those of their affiliated organizations, or those of the publisher, the editors and the reviewers. Any product that may be evaluated in this article, or claim that may be made by its manufacturer, is not guaranteed or endorsed by the publisher.
